# In Situ Formation of a Relatively Transparent Ion-Associate Liquid Phase from an Aqueous Phase and Its Application to Microextraction/High-Performance Liquid Chromatography–Fluorescence Detection of Bisphenol A in Water

**DOI:** 10.3390/molecules28227525

**Published:** 2023-11-10

**Authors:** Noriko Hata, Seira Takahashi, Sachiko Osada, Sakura Katagiri, Mayumi Naruse, Akane Igarashi, Kazuto Sazawa, Shigeru Taguchi, Hideki Kuramitz

**Affiliations:** Major of Earth, Life and Environmental Science, Graduate School of Science and Engineering, University of Toyama, 3190 Gofuku, Toyama 930-8555, Japansazawa@sci.u-toyama.ac.jp (K.S.); crewoftaguchiut@gmail.com (S.T.); kuramitz@sci.u-toyama.ac.jp (H.K.)

**Keywords:** in situ solvent formation, microextraction, ion-associate liquid phase, ethylhexyloxypropylamine, dodecylsulfate, bisphenol A, HPLC-fluorescence detection

## Abstract

The design of a simple approach enabling the detection of bisphenol A (BPA) in water samples without the need for large amounts of solvents is of utmost importance. This paper reports a simple method for the separation, concentration, and quantification of BPA in water samples using high-performance liquid chromatography with fluorescence detection (HPLC-FLD) after its microextraction into an in situ formed organic ion-associate (IA) liquid phase (LP). Novel IA phase components without conjugated double bonds, such as benzene rings, were investigated. Ethylhexyloxypropylamine hydrochloride and sodium dodecyl sulfate solutions were added to the water samples to form IAs. The aqueous phase and ion-associate liquid phase (IALP) were separated by centrifugation. The aqueous phase was removed, and the liquid phase was recovered and measured using HPLC-FLD or HPLC-electrochemical detection (ECD). The concentrated phase (IALP) had a relatively low viscosity and could be injected directly into the chromatograph without dissolving it in organic solvents. The detection limits for BPA by HPLC-FLD and HPLC-ECD were 0.009 and 0.3 µg L^−1^, respectively.

## 1. Introduction

In the analysis of trace constituents in environmental water samples, a sample pretreatment step, such as concentration and separation, is often necessary to improve the sensitivity and selectivity of analytical techniques [1,2,3,4]. 

Solvent extraction is one of the most commonly used methods for the separation and concentration of environmental pollutants. However, the major drawbacks of conventional extraction methods include the (1) difficulty to increase the extractant concentration because of its partition between the two solvents, (2) toxicity of some extraction solvents such as chloroform, dichloromethane, and benzene [5,6] (whose concentrations in wastewater were regulated in 1993) [7], and (3) need for shaking and mixing to promote partitioning between the two phases [8,9]. 

Recently, the employment of green analytical approaches such as microextraction, which enables the extraction of a high concentration of a target substance in small amounts, has been gaining attention. For example, dispersive liquid–liquid microextraction [10,11] involves the use of a vortex or ultrasound to promote partitioning between the two phases [11] or the addition of 1/8 of the sample water volume of the dispersing solvent [3]. In this technique, a very small amount of a mixture of hydrophobic extraction and dispersing solvents is injected into the sample solution to extract the target component in a microvolume phase. Furthermore, some microextraction methods, such as homogeneous liquid–liquid extraction (HLLE) [12], cloud point (CP) extraction [13,14,15], ionic liquid [16], deep eutectic solvent [9,10,17,18], and our proposed organic ion-associate liquid phase (IALP) extraction, can enable in situ solvent formation. HLLE employs organohalogen compounds such as perfluorooctanoic acid in which the analyte is extracted from a homogeneous solution into a very small sedimented phase by pH modification, heating, salt addition (salting out), and constituent mixing/replacement [12]. CP extraction uses the cloud point of nonionic surfactants to separate the surfactant micellar phase from the aqueous phase to concentrate hydrophobic substances [13,14,15] upon heating.

In IALP microextraction [19,20,21,22,23,24], organic cations and anions are added to a water sample to form the IALP. The IA liquid and aqueous phases are then separated by centrifugation to extract the target component into the liquid phase. The target component is then dissolved in an organic solvent in the IALP or back-extracted from the IALP into an acid for measurement. This approach features several advantages, including the high capacity of the extractant, rapid extraction, avoiding harmful organic solvents, and minimization of contamination because it can be performed in a single container. Several studies have reported the application of this method for the separation, enrichment, and detection of ammonium ion, di(2-ethylhexyl) phthalate, cadmium, etc. [19,20,21,22,23,24]. 

Bisphenol A (BPA) is widely used as a raw material in the production of epoxy resin and polycarbonate plastics. In 2019, the total domestic production of BPA in Japan amounted to approximately 460,000 t [25]. However, leaching of BPA can occur through the hydrolysis of the polymer. High temperatures can increase this leaching [25,26,27,28,29,30,31]. Furthermore, it has been included in the Candidate List of Substances of Very High Concern according to the European Registration, Evaluation, Authorization, and Restriction of Chemicals regulations [26]. Japan currently requires a BPA content in water lower than 0.1 mg L^−1^ [27]. The effects of BPA on human health have already been reported [30]. Li et al. have also reported the detection of BPA in human urine [32]. Moreover, there is an increasing concern regarding the effects of BPA in environmental water on aquatic organisms [31]. Therefore, it is important to monitor BPA in environmental water. Consequently, the quantification of BPA is of great significance.

The analytical approach employed by the Ministry of the Environment for BPA detection [29] involves the acidification of a water sample (or filtrate) followed by extraction with dichloromethane (or performing solid-phase extraction and subsequent elution with dichloromethane). The extract is then dehydrated and concentrated for trimethylsilylation prior to its measurement by GC/MS-selected ion monitoring (SIM) mode. This approach not only requires large volumes of the water sample and extraction solvent, but also involves complex procedures, requiring derivatization for measurement.

Based on the Japanese and WHO guidelines, the maximum acceptable concentration of dichloromethane is 0.02 mg L^−1^ [5,6]; however, the solubility of dichloromethane in water [33] is one million times higher than this value. Consequently, care must be taken when handling the effluents.

IALP extraction methods often utilize certain reagents (dye-forming reagents [19], chelating reagents [22,23], pH buffers [21]) to convert the target component into an appropriate form (dye [19], chelate [22,23]), which is then added to the IALP with the counter ions. Hata et al. reported the extraction of polyaromatic hydrocarbons (PAHs) by an IALP composed of a combination of benzethonium (Ben^+^) chloride and ethylbenzenesulfonate sodium salt without conversion prior to their detection by high performance liquid chromatography with fluorescence detection (HPLC-FLD) [24]. However, the influence of organic reagents and their impurities on the IALP rendered the detection of light PAHs with three or fewer rings difficult. Although the combination of organic ions constituting a conventional IALP allowed for a high enrichment factor, their retention times in HPLC/FLD were relatively close to that of the hydrophilic analyte species (BPA), which interfered with the measurements.

So far, all organic ions selected for the IALP have benzene rings and exhibit fluorescence [19,20,21,22,23,24]. Consequently, it is necessary to find IALP components without benzene rings or imidazole groups among organic ions to develop an IALP that can be used for the separation and concentration of BPA. 

This study reports a rapid, simple, and environmentally friendly analytical method for monitoring BPA in water by HPLC-FLD and HPLC with electrochemical detection (HPLC-ECD) following IALP extraction. 

The IALP extraction method has the following advantages: (1) various types of organic cations and anions can be selected according to the application and can be monitored using various types of equipment; (2) a high enrichment factor can be obtained; (3) the target component can be extracted quickly because the extractive phase is precipitated from the aqueous phase; and (4) the method can be performed in a single vessel, thus minimizing contamination. 

## 2. Results

### 2.1. Organic Ions Constituting the Ion-Associate Liquid Phase (IALP)

#### 2.1.1. HPLC-FLD

The individual extraction constants for the organic anions (log *K*_anion_, [34]) and octanol–water partition coefficients (log *K*_owwin_, [35]) of the organic substances considered in this investigation are listed in Table 1. The individual extraction constant for anions (log *K*_anion_) was obtained by separating the extraction constant (log *K*_ex_) for ion-pair extraction into the respective contributions from cations, anions, and the extraction solvent. 

**Figure 1:**
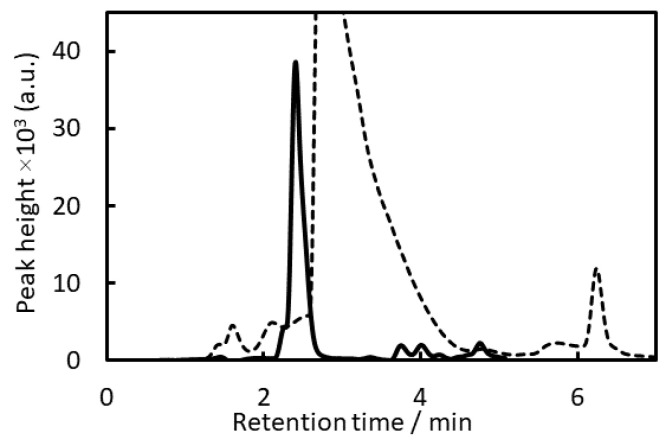
HPLC-FLD chromatograms. Solid line: bisphenol A (BPA) after microextraction of IALP by (ethylhexyloxypropylamine (EHOPA^+^)/dodecylsulfate (DS^−^)); BPA concentration: 2 µg L^−1^; dotted line: IALP (benzethonium ions (Ben^+^)/Ethylbenzenesulfonate ion (EBS^−^)).

HPLC-FLD chromatograms of BPA after microextraction of IALP by EHOPA^+^/DS^−^ or IALP (Ben^+^/ EBS^−^) alone are shown in Figure 1. 

#### 2.1.2. HPLC-ECD

Electrochemical detection is a method for studying compounds that undergo redox reactions; examples include compounds with phenolic hydroxyl groups. Four pairs of organic cations and organic anions forming the IALP were combined: 4-trifluoromethylanilium ion and dodecylbenzenesulfonate ion (DBS^−^), benzyldimethyldodecylammonium ion and phenol sulfonate ion (PS^−^), Ben^+^ and PS^−^, and Ben+ and ethylbenzenesulsonate (EBS^−^). The peaks of the reagent and the impurities that generated the IALP overlapped with the BPA peak, which was not desirable. Since EBS^−^ and Ben^+^ should not be detectable by ECD under the conditions of this experiment, they were considered to be derived from the impurities. Therefore, when EBS^−^ and Ben^+^ were recrystallized, the impurities in EBS were considerably reduced, but the impurities derived from Ben^+^ could not be removed. The combination of IALPs developed for FLD, EHOPA^+^, and DS^−^ was also investigated for use in the ECD of BPA. As a result, the concentrates produced by the IALPs (EHOPA^+^ and DS^−^) could be used to detect BPA by HPLC-ECD.

### 2.2. Regions of Ion-Associate (IA) and Liquid Phase (LP) Formation 

**Figure 2:**
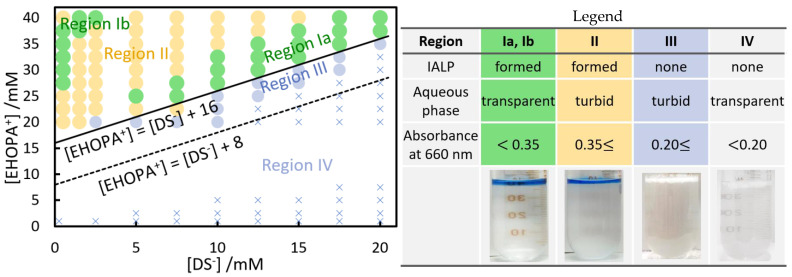
Effect of EHOPA^+^ and DS^−^ concentrations on IALP formation. Experimental condition: [EHOPA^+^] = 0.5 × 10^−3^ to 40 × 10^−3^ mol L^−1^, [DS^−^] = 0.5 × 10^−3^ to 20 × 10^−3^ mol L^−1^. After centrifugation, the standing time was 1 h; temperature during IA formation: 25 °C. Photographs of regions I and II represent IALP colored by adding bromophenol blue dye in base type.

The concentrations of EHOPA^+^ and sodium dodecyl sulfate (SDS) during the formation of IAs and solvents were investigated by varying their concentrations in two dimensions (Figure 2). 

### 2.3. Effect of Organic Ions on Ion-Associate Liquid Phase (IALP) Volume, Percent Extraction (%E), and Distribution Constant (K_d_)

**Figure 3:**
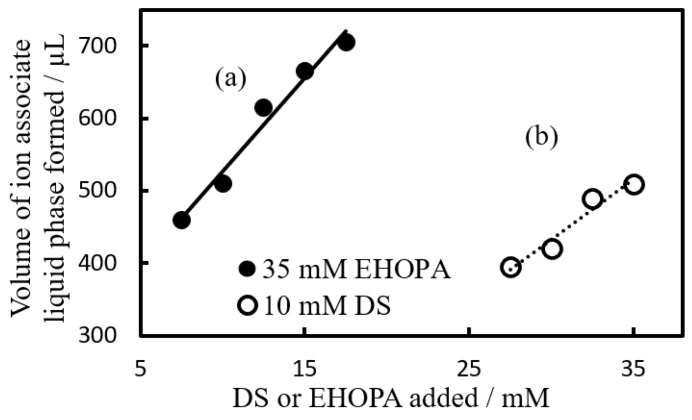
Effect of constituent organic ions on the volume of IALP. (a) Black circle: [EHOPA^+^] = 35 × 10^−3^ mol L^−1^, [DS^−^] = 7.5 to 17.5 × 10^−3^ mol L^−1^. (b) White circle: [EHOPA^+^] = 27.5 to 35 × 10^−3^ mol L^−1^, [DS^−^] = 10 × 10^−3^ mol L^−1^. After centrifugation, the standing time was 1 h.

Figure 3a shows the case where EHOPA^+^ concentration was held constant (35 mM) while DS^−^ concentration varied, and Figure 3b shows the case where [DS^−^] was 10 mM while EHOPA^+^ concentration varied. 

**Figure 4:**
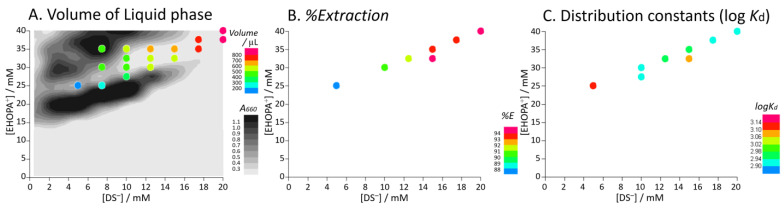
Relationship between EHOPA and DS concentrations on the volume of ion-associate liquid phase (IALP), %*E* of BPA, and distribution coefficient (log *K*_d_) of BPA. Experimental conditions: [EHOPA^+^] = 25 × 10^−3^ to 40 × 10^−3^ mol L^−1^, [DS^−^] = 5 × 10^−3^ to 20 × 10^−3^ mol L^−1^, [BPA] = 5.0 µg L^−1^.

Figure 4 shows the volume of the IALP formed, %*E* of BPA, and the partition constant for each concentration. The absorbance (turbidity) at 660 nm is shown in a grayscale contour plot (Figure 4A). As the concentration of EHOPA or DS increased, IAs were formed, resulting in a cloudy sample. Further increasing the concentration resulted in the formation of a liquid phase which became transparent in the appropriate concentration range, while IAs were colloidally dispersed and become cloudy in other regions. As shown in Figure 4A, the volume of the liquid phase increased upon increasing the concentrations of EHOPA and DS. 

### 2.4. Analytical Figure of Merit

The detection limit 3σ_b_ (n = 3), enrichment factor, %*E*, and log *K*_d_ were determined for different concentrations of EHOPA^+^ and DS^−^. The results are summarized in Table 2. At an EHOPA^+^ concentration of 27.5 mM and DS^−^ concentration of 10 mM, the detection limit of BPA was 0.049 µg L^−1^, %*E* was 89%, and log *K*_d_ was 2.9. At an EHOPA^+^ concentration of 25 mM and DS^−^ concentration of 5 mM, the detection limit of BPA was 0.009 µg L^−1^, %*E* was 86 %, and log *K*_d_ was 3.1. For HPLC-ECD, at an EHOPA^+^ concentration of 31 mM and DS^−^ concentration of 12 mM, the detection limit of BPA was 0.2 µg L^−1^, %*E* was 93%, and log *K*_d_ was 3.0. Enrichment factors were calculated by comparing the slope of the calibration curve of the standard solution with and without microextraction; because the volumes of IALP were different, the enrichment factor by IALP microextraction was 150–300-fold. As shown in Figure 4B,C, %*E* and log *K*_d_ obtained by HPLC-FLD ranged from 86% to 95% and 2.9 to 3.1, respectively, and %*E* and log *K*_d_ obtained by HPLC-ECD were also within these ranges and could be considered reasonable values.

Table 3 lists the limits of detection of some previously reported methods. Our proposed method has the lowest limit of detection. 

### 2.5. Application to River Water Samples

**Figure 5:**
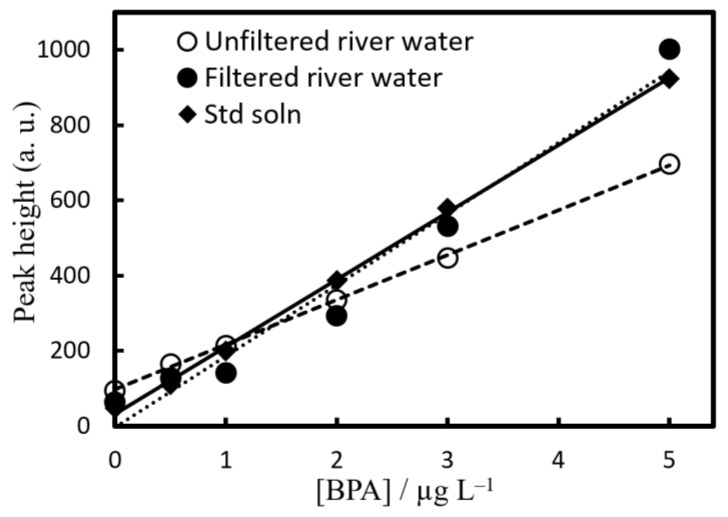
Calibration curves of BPA in pure water (◆) and river water sample (○,●). Concentration of BPA was 0–5 µg L^−1^. Experimental conditions: [EHOPA^+^] = 25 × 10^−3^ mol L^−1^, [DS^−^] = 5 × 10^−3^ mol L^−1^, pH = 1.9. (**○**) Nonfiltered water and (**●**) filtered water.

To validate this method, quantification and recovery experiments were conducted on river water samples (Jinzu River (Toyama Prefecture), Hagiura Bridge; pH 6.54, EC 45.8 mS m^−1^) using EHOPA^+^/DS^−^ = 25 mM/5 mM. For recovery experiments, river water samples containing 0.5, 1, 2, 3, and 5 µg L^−1^ BPA were also prepared (Figure 5). 

## 3. Discussion

All previously reported organic ions used for IALP extraction have benzene rings, and their retention times and those of their impurities overlap with those of BPA. Thus, if an IALP is composed of Ben^+^Cl^−^ and EBS^−^, BPA cannot be detected because the peaks of the organic ions forming the IALP and the impurities will overlap with those of BPA (Figure 1). Therefore, in this study, we investigated organic ions without benzene rings but with low fluorescence intensities for the HPLC-FLD of BPA after IALP extraction. 

In ion-pair extraction, the extraction constant, *K*_ex_, includes the formation of ion pairs (*K*_ass_) and the distribution of the ion pairs formed (*K*_d_); *K*_ow_ (*K*_owwin_), often used as a measure of the hydrophobicity of a substance, is only related to its distribution and not to the formation of ion pairs. Individual extraction constants for anions (log *K*_anion_) [34] are used as references to select the optimum organic anions. Individual extraction constants are the extraction constants for ion-pair extraction (log *K*_ex_) separated into log *K*_cation_, log *K*_anion_, and log *K*_solvent_. Extraction constants represent the ease of association of cations and anions and the ease of distribution between the aqueous phase and extraction solvent and are considered helpful in selecting organic anions for the IALP. DBS^−^ [20] is the only anion studied so far for which log *K*_anion_ has been determined among the anions constituting the IALP. Therefore, we selected organic anions with log *K*_anion_ similar to that of DBS, and their log *K*_anion_ and *K*_owwin_ are listed in Table 1. Of the three organic anions without benzene rings, SS^−^, DS^−^, and tetradecyl sulfonate (C_14_S^−^), DS^−^ has the lowest *K*_owwin_ but the same log *K*_anion_ as that of DBS^−^. Furthermore, DS^−^ was chosen as the organic anion because it is often used in the field of biochemistry and can be obtained in high purity. 

Dodecyltrimethylammonium bromide, tetradecyltrimethylammonium bromide, and Zephiramine (Zeph^+^, benzyldimethyltetradecylammonium chloride) were examined as counter ions for detecting indothymol, which is more hydrophobic than BPA. However, only Zeph^+^ could quantitatively recover indothymol in the case of collection on a glass fiber filter [37]. Cetyltrimethylammonium chloride is not very soluble in water. Therefore, these alkyltrimethylammonium were not considered. In this study, amines were protonated to ammonium salts, which were considered as organic cations for IALP formation. Many peaks due to impurities were observed in previous IALPs. In general, commercially available reagents, including analytical grade reagents, contain a small amount (a few percent) of impurities. Amines were chosen because they can usually be purified easily by changing the pH and can be used for back-extraction.

The following reagents were thus selected as candidates for organic cations and attempts were made to prepare aqueous solutions for each reagent: Bis[3-(trimethoxysilyl)propyl]amine instantly solidified when mixed with water, while 3-butoxypropylamine (BOPA) and tris[2-(2-methoxyethoxy)ethyl]amine (TMOEEA) were soluble in water. N-methyldidodecylamine (MDDA) and EHOPA^+^ did not dissolve in water. EHOPA^+^ dissolved in hydrochloric acid, while MDDA did not. Consequently, TMOEEA, BOPA, and EHOPA were chosen as the organic cationic reagents, while DS^−^, which is soluble in water, was considered as the organic anionic reagent. The TMOEEA, BOPA, and EHOPA HCl (EHOPA^+^) solutions, in addition to DS^−^, were used to obtain samples with varying cation and anion concentrations. When the obtained samples were cloudy, they were centrifuged and subsequently observed for the formation of IALP. In the combination of TMOEEA and DS^−^, their respective concentrations varied from 2.5 to 20 mM, while they varied from 2.5 to 25 mM in the combination of BOPA and DS^−^. No IALP was obtained with these two combinations. The aqueous phase could be separated from the IALP only when EHOPA^+^ was used as an organic cation. Based on these results, the EHOPA^+^/DS^−^ combination was selected as the organic ion constituting the IALP in this study. The formed IALP floated above the aqueous phase. 

In the analysis of BPA, it is common to make water samples acidic to prevent BPA dissociation; however, in this method, the pH is set to 2 by adding EHOPA hydrochloric acid solution [29]. Thus, it is not necessary to make the water particularly acidic.

In Region I (Figure 2), an IALP was formed at the top and the aqueous phase was clear (absorbance at 660 nm was less than 0.35). In Region II, an IALP was also formed, but the aqueous phase was cloudy (absorbance at 660 nm was greater than 0.35). In Region III, the aqueous phase was cloudy (absorbance at 660 nm was greater than 0.35), but no IALP was formed. In Region IV, the aqueous phase was clear (absorbance at 660 nm was less than 0.15). The IALP formed from Ben^+^Cl^−^ and EBS^−^ had a single Region I [24], while for the EHOPA^+^/DS^−^ combination, Region I was divided into two Regions, Ia and Ib (Figure 2). The volume of the IALP formed in Region Ib was smaller than that in Region Ia. The equations are:

[EHOPA^+^] = [DS^−^] + 8 mmol L^−1^, (1)

[EHOPA^+^] = [DS^−^] + 16 mmol L^−1^, (2)

The critical micelle concentration (CMC) of SDS is 16.9 mmol L^−1^ [38]. For an IALP to form, there must be an EHOPA^+^ concentration higher than the concentration of DS^−^ plus 16 mmol L^−1^. 

The results (Figure 3 and Figure 4A) revealed that the IALP increased as the amount of added organic ions increased. 

As seen in Figure 3, the effect of the organic anions on the volume of the IALP formed was approximately 1.5 times greater than that of the organic cations. The volume of the IALP was more influenced by the concentration of DS^−^ than by the concentration of EHOPA^+^.

In Figure 4A, the volume of the IALP increases with increasing concentrations of EHOPA and DS, i.e., the volume of the IALP is affected by EHOPA and DS concentrations. In the grayscale contour plot in Figure 4A, at [DS] = 5 mM, as the concentration of EHOPA increases, IAs form and the solution becomes turbid (Region IV →Region III). After centrifugation, at [EHOPA] = 25 mM, an LP is formed and turbidity decreases (Region Ia). Further, as EHOPA increases, some of the IAs formed aggregate to form the LP, while some disperse and become turbid (Region II).

In order to evaluate the ability of the IALP (EHOPA^+^/DS^−^) to extract IAs, the distribution constant of BPA was determined: the *K*_ow_ of BPA is log *K*_owwin_ = 3.64 [35]. The *K*_d_ of BPA in the IA phase can be expressed as follows:

(3) $$K_{d}=\frac{{\left[\mathrm{BPA}\right]}_{\mathrm{ialp}}}{{\left[\mathrm{BPA}\right]}_{\mathrm{aq}}}$$

Equation (3) was transformed to obtain the *K*_d_.

(4) $$K_{d}=\frac{m_{\mathrm{ialp}}\;/\;v_{\mathrm{ialp}}}{m_{\mathrm{aq}}/\;v_{\mathrm{aq}}}=\frac{v_{\mathrm{aq}}}{v_{\mathrm{ialp}}}\cdot \frac{m_{\mathrm{ialp}}}{m_{\mathrm{aq}}}$$

Here, *m* is the amount of the substance, *v* is the volume, _ialp_ is the IALP, and _aq_ is the aqueous phase. The amount of the substance was calculated by determining the percentage extraction of BPA at each concentration. The volume of the aqueous phase was calculated by subtracting the volume of the IALP.

As described in a previous study [24], a calibration curve was constructed from the peak heights of the chromatograms of the first and second extractions, and %*E* was calculated from the slope of the curve. If the slope at 100% recovery is α, %*E* can be obtained from the slopes of the calibration curves of the first and second extractions. The calibration curves for BPA from the IALP (EHOPA^+^/DS^−^) extraction (first extraction) and the calibration curve obtained by extracting its aqueous phase again with IALP (second extraction) are shown in Figure S1.

(5) $$\mathrm{Slope}\;\mathrm{of}\;\mathrm{the}\;\mathrm{first}\;\mathrm{extraction}=\alpha \;\times \;\frac{\%E}{100}$$

(6) $$\mathrm{Slope}\;\mathrm{of}\;\mathrm{the}\;\mathrm{second}\;\mathrm{extraction}=(\alpha \;\times \;\frac{100-\%E}{100}\;)\;\times \;\frac{\%E}{100}$$

In Figure 4B, %*E* of the IALP increases with increasing concentrations of EHOPA and DS, i.e., %*E* of the IALP is affected by the concentrations of EHOPA and DS. The IALP/water log *K*_d_ was not significantly affected by the concentrations of EHOPA and DS (Figure 4C). The IALP/water log *K*_d_ ranged from 2.9 to 3.1 which was slightly smaller than the octanol–water partition constant, log *K*_ow_ = 3.32 [35], indicating that the IALP (EHOPA^+^/DS^−^) had a slightly smaller extraction ability for BPA than octanol. For each concentration condition, %*E* ranged from 87.6% to 94.7%, where the %*E* increased as IALP volume increased. When EHOPA and DS were added, the pH of the aqueous phase was approximately 2 since EHOPA was dissolved in HCl (2 M).

The fact that our proposed method showed the lowest detection limit among the methods listed in Table 3 may be due to its high concentration factor and good reagent purity as well as owing to the fact that the concentration step was performed in a single vessel.

When a recovery test was performed using unfiltered river water samples, the slope was slightly lower than that of the calibration curve of ultrapure water. Therefore, an additive recovery test was performed using a river water sample filtered through a glass fiber filter. The results were in agreement with the slope of the calibration curve of the standard solution, thus indicating that the slope was reduced because of the influence of suspended solids in the river water sample.

The BPA content in the water of the Furu River, a small tributary of the Jinzu River, measured by HPLC-ECD was below the detection limit. Therefore, we conducted an additive recovery experiment to verify whether BPA could be recovered quantitatively. In the addition recovery experiment, BPA was added to water at concentrations of 5, 10, and 15 nM for 40 mL. The recovery obtained from the slope was 90%, indicating that quantitative addition and recovery were possible for river water samples. In this IALP combination, a good linear calibration curve was obtained in the range of 5 to 15 nM, and the detection limit (3σ) was 0.3 μg L^−1^. In addition, when the recovery experiment was applied to environmental water samples, the recovery was as high as 90%.

## 4. Materials and Methods

### 4.1. Reagents and Chemicals

**Figure 6:**
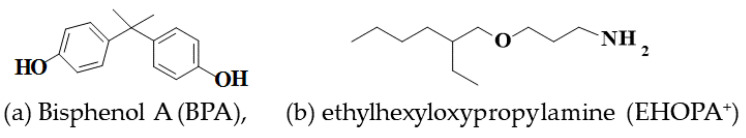
Structure of bisphenol A (BPA) and EHOPA.

The tris[2-(2-methoxyethoxy)ethyl]amine [organic cation] solution (1 M) was prepared by dissolving tris[2-(2-methoxyethoxy)ethyl]amine (Tokyo Chemical Industry, Tokyo, Japan) (32 g) in water (100 mL). The 3-butoxypropylamine [organic cation] solution (0.5 M) was obtained by dissolving 3-butoxypropylamine (6.6 g, Tokyo Chemical Industry, Japan) in water (100 mL). The EHOPA^+^Cl^−^ solution was prepared by dissolving ethylhexyloxypropylamine (18.7 g, Tokyo Chemical Industry, Japan) with a HCl solution (2 M, 100 mL). The sodium dodecyl sulfate [organic anion] solution (0.1 M) was prepared by dissolving sodium lauryl sulfate (2.9 g, specially prepared reagent for biochemical research, Nacalai Tesque Inc., Kyoto, Japan, purity ≥ 99.5% (as C12, GC)) in water (100 mL). A standard BPA solution (100 mg L^−1^) was prepared by dissolving BPA (10 mg, Wako Pure Chemical, Osaka, Japan) in acetonitrile (100 mL). The HCl solution (2 M) was prepared by mixing HCl and water at a volume ratio of 1:5. The tetramethylammonium hydroxide (5%, TMAH) aqueous solution was prepared by diluting TAMAPURE AA100 TMAH (10 mL, 25 %, Tama Chemical, Kawasaki, Japan) up to 50 mL using water. The TMAH ethanol solution (5%) was prepared by diluting TAMAPURE AA100 TMAH (10 mL, 25%) to 50 mL using ethanol. Figure 6 shows the structure of BPA and the cation source, EHOPA. 

All other reagents were of analytical grade and used as received. In all experiments, ultrapure water (18.2 MΩ) made by an ultrapure water production system (Direct-Q3UV (Millipore, Burlington, MA, USA)) was used.

Water samples were stored either in brown glass bottles or polyethylene bottles and kept in a cool, dark place. 

### 4.2. Apparatus

The UV-VIS spectra were recorded with a Shimadzu Model UV-2450 spectrophotometer using a 1 cm quartz cell or a 1 cm quartz cell with a 2 mm optical path width. A centrifuge (Type 5420, Kubota, Tokyo, Japan) was used for centrifugation. A thermominder system (SX-10N; Taitec, Koshigaya, Japan) was used as a thermostatic apparatus. pH measurements were carried out using a D-24 pH meter (Horiba, Kyoto, Japan). 

### 4.3. Ion-Associate (IA) and Liquid Phase (IALP) Formation

Water (~30 mL) was added to a glass centrifuge tube (50 mL). The organic cation (EHOPA^+^, 1 M, 0.8 to 1.6 mL), HCl solution (2 M), and organic anion (DS^−^, 0.1 M, 0.2 to 8 mL) solution were then added, respectively. Finally, the volume was made up to 40 mL by adding water, and the solution was mixed. The aqueous phase was visually checked for the formation of IAs (turbidity or transparency) and centrifuged (3500 rpm for 30 min). After a standing time of 1 h, the formation of the IALP and the conditions of the aqueous phase were visually examined. The turbidity of the aqueous phase after centrifugation was determined by measuring absorbance at 660 nm using a UV-vis spectrophotometer. Temperature was maintained at 25 °C using a thermostatic device when studying the conditions for IALP formation since DS^−^ precipitates at low temperatures. 

### 4.4. Ion-Associate Liquid Phase (IALP) Microextraction 

The water sample (40 mL) along with EHOPA^+^ HCl (1 M) and Na^+^DS^−^ (0.1 M) solutions were centrifuged for 30 min to separate the IA liquid and aqueous phases. The aqueous phase was then removed. After centrifugation for 1 min to remove the water droplets adhering to the vessel wall, the IALP was directly collected and measured by HPLC-FLD or HPLC-ECD). 

BPA was detected by reverse-phase HPLC, followed by two different detectors: FLD and ECD. The FLD of BPA was performed using a Prominance simple system (LC-20AT, RF-10AXL, DGU-20 A3, LC workstation, Shimadzu, Japan), an injection valve with a 20 μL loop (Rheodyne, Rohnert Park, CA, USA), and an Inertsil ODS-3 chromatography column (4.0 mm I.D. × 150 mm, 3 μm, GLSciences, Japan; column temperature: 40 °C, flow rate 0.7 mL min^−1^). The eluent was prepared by mixing acetonitrile and a potassium chloride solution (0.05 M) at a ratio of 75:25. For the ECD of BPA, an HPLC system (system controller: SCL-10Avp, data processing, column oven, Shimadzu damper, DGU-12A, Shimadzu, Kyoto, Japan) with a C18 reverse-phase column (CAPCELL PAK C18 MG II HPLC column (150 × 4.6 mm, 3 μm, Shiseido, Kyoto, Japan) or Inertsil ODS-3 chromatography column (see above)) and an injection valve (Rheodyne, USA) with a 20 μL loop was used. BPA was detected using a coulometric detection system (ECD, Guard Cell Model 5020, Analytical Cell Model 5010, ESA, Memphis, TN, USA) consisting of two porous carbon working electrodes controlled using a Coulochem III (ESA, USA) potentiostat. The eluent consisted of phosphate buffer (50 mM, pH 2.1)/acetonitrile (50:50) mixture. The rate of the mobile phase flow through the system was 0.5 mL min^−1^. The initial potential was 650 mV and the measured voltage was 800 mV.

**Figure 7:**
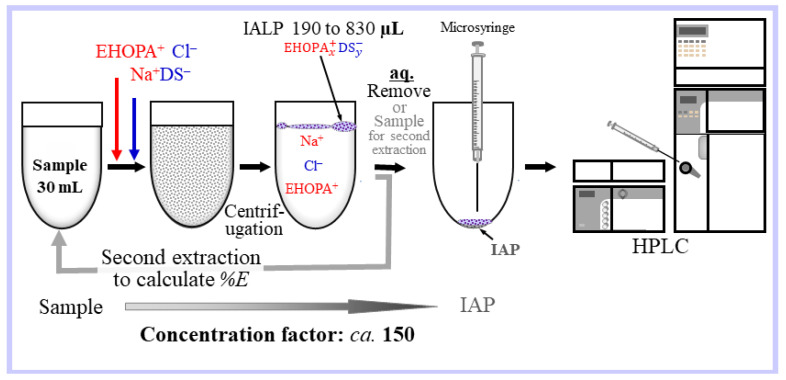
Schematic of the microextraction of an IALP formed in situ from the aqueous phase by adding EHOPA, HCl, and SDS.

A schematic of the microextraction of an IALP generated in situ from the aqueous phase by adding the EHOPA^+^, HCl, and Na^+^DS^−^ solutions is presented in Figure 7. For the preparation of the IALP, EHOPA (1 M) and DS^−^ (0.1M) were added to the water sample (40 mL) in a glass centrifuge tube (50 mL) to form an IA emulsion to extract the BPA into the IALP. After centrifugation at 3500 rpm for 30 min, the IALP and aqueous phase were separated. The IALP from which BPA was extracted was taken with a syringe, injected, and analyzed using HPLC-ECD.

For the second extraction, the aqueous phase was transferred to another centrifuge tube and DS^−^ (0.1 M) was added to form another IALP. The solution was centrifuged to separate the (second) IALP from the aqueous phase. The concentrate (IALP) was injected directly into the HPLC system and analyzed by FLD or ECD, and the %*E* of BPA from the IALP consisting of EHOPA^+^DS^−^ was determined.

### 4.5. Measurement of IALP Volume

Water (~30 mL) was added into a glass centrifuge tube (50 mL). EHOPA^+^ and DS^−^ were then added, followed by the addition of water, to achieve a final volume of 40 mL. The mixture was then centrifuged (30 min, 3500 rpm) to separate the IA liquid and aqueous phases. The aqueous phase was removed. The mixture was centrifuged (3 min, 3000 rpm) again to collect the aqueous phase adhering to the vessel wall and remove the collected aqueous phase. Volume was measured by aspirating the IALP using a microsyringe and reading the scale.

## 5. Conclusions

This study employed fluorescent organic ions in an IALP extraction method. An IALP formed from EHOPA^+^DS^−^ enabled the quantitative extraction of BPA, an endocrine-disrupting chemical with a predicted no-effect concentration in environmental water of 11 μg L^−1^ [27], prior to its successful detection by HPLC-FLD and HPLC-ECD. The developed method is environmentally friendly and can be used as a pretreatment for the separation and concentration of other fluorescent and electrochemically reactive substances such as PAHs and estrogens prior to HPLC. 

This method enabled a reduction in sample volume to approximately 1/20 of that required using the method employed by the Ministry of the Environment. The analysis time was also reduced, from 3 h to less than 1 h. A sufficient quantitative range was obtained for the concentrations required for measurement. Furthermore, dichloromethane, a hazardous chemical, was not used in this study. The concentrate was prepared in a single centrifuge tube without changing the instrument and had a low viscosity. It was directly collected using a microsyringe and injected into the HPLC system. The concentrates were sufficiently clear for both FLD and ECD. Consequently, the proposed method can be considered an efficient and environmentally friendly detection method.

## Figures and Tables

**Table 1:** Organic substances and their log *K*_anion_ and log *K*_owwin_ values.

Organic Substances		log *K*_anion_ [34]	log *K*_owwin_ [35]	Molecular Weight
Name (Abbreviation)	Formula			
Organic anions			as sodium salts	
Bis(2-ethylhexyl) Sulfosuccinate (SS^−^)	(C_8_H_17_COO)_2_C_2_H_3_SO_3_^−^	2.2	3.95	444.56
Dodecylbenzenesulfonate (DBS^−^)	C_12_H_25_C_6_H_4_SO_3_^−^	1.5	3.00	348.48
Tetradecylsulfonate (C_14_S^−^)	C_14_H_29_SO_3_^−^	0.9	1.86	300.44
Dodecylsulfate (DS^−^)	C_12_H_25_SO_4_^−^	1.5	1.69	288.38
Substances for organic cations and analyte				
Tris[2-(2-methoxyethoxy)ethyl]amine	(CH_3_OC_2_H_4_OC_2_H_4_)_3_N	--	−1.61	323.43
3-Butoxypropylamine	C_4_H_9_OC_3_H_6_NH_2_	--	1.05	131.22
Ethylhexyloxypropylamine (EHOPA^+^)	C_11_H_25_NO	--	2.94	187.33
N-Methyldidodecylamine	(C_12_H_25_) _2_NCH_3_	--	10.84	367.7
BPA	C_15_H_16_O_2_	--	3.64	228.29

**Table 2:** Limit of detection (LOD), %*E*, log *K*_d_, and enrichment factor for different EHOPA^+^ and DS^−^ concentrations.

Detection	[EHOPA^+^]/mM	[DS^−^] /mM	LOD		%*E*	log *K*_d_	Enrichment Factor
			/µg L^−1^	nM			
Fluorescence	27.5	10	0.049	0.21	89	2.9	290
	25	5	0.009	0.04	86	3.1	310
Electrochemical	31	12	0.2	1	93	3.0	150

**Table 3:** Liquid and solid phase extraction procedure for bisphenol A.

Sample Preparation	Analysis Method	Sample or Matrix	Extractant	LR/µg L^−1^	LOD/µg L^−1^	Enrichment Factor	Reference
VALLME	HPLC-FLD	natural water	2-ethylhexanol	0.1–100	0.02	--	[8]
CPE	HPLC-UV	river water	AEO9, octanol	0.05–20	0.27	--	[14]
DLLME	CE	tap water, lake water, seawater	chlorobenzene	4–300	0.6	241	[3]
UTA-CPE	UV–vis spectrophotometry at 643 nm	drinking water	Brij 35	1.2–160	0.35	180	[15]
SPE	UHPLC-MS/MS	urine	mixed-mode anion-exchange SPE	0.5–50	0.13	--	[32]
VALLME	HPLC-FLD	plastic materials	hydrophobic des (decanoic acid, trioctylmethyl ammonium chloride)	0.3–700	0.06	110.3	[9]
SUPRAS-ME	Automated HPLC-FLD	beverages	supramolecular solvent (1-hexylamine, menthol)	2–5000	0.7	10.4	[36]
IALPME	HPLC-FLD	water	ethylhexyloxypropylamine, dodecylsulfate	0.5–5	0.009	310	This work

LR: linear range; LOD: limit of detection; VLLME: vortex-assisted liquid–liquid microextraction; HPLC: high-performance liquid chromatography; FLD: fluorescence detection; CPE: cloud point extraction; AEO9: alcohol ethoxylate; DLLME: dispersive liquid–liquid microextraction; CP: capillary electrophoresis; UTA-CPE: ultrasonic-thermostatic-assisted cloud point extraction; UHPLC-MS/MS: ultra-high-performance liquid chromatography–tandem mass spectrometry; Brij 35: polyethylene glycol dodecyl ether; HPLC-UV: HPLC with UV detection; des: deep eutectic solvents; IALPME: ion-associate liquid phase microextraction.

## Data Availability

The data are contained within the article and its supporting information.

## Supplementary Materials

The following supporting information can be downloaded at: https://www.mdpi.com/article/10.3390/molecules28227525/s1, Figure S1: Calibration curve for bisphenol A (BPA) from the first and second extractions from IALP (EHOPA^+^/DS^−^).
