# Supplementary material for: In Situ Formation of a Relatively Transparent Ion-Associate Liquid Phase from an Aqueous Phase and Its Application to Microextraction/High-Performance Liquid Chromatography–Fluorescence Detection of Bisphenol A in Water
Source: Molecules. 2023 Nov 10;28(22):7525. doi: 10.3390/molecules28227525 (PMC10673447; doi:10.3390/molecules28227525)
Supplement: Supplementary file 1 [file molecules-28-07525-s001.zip › molecules-2673809-supplementary.pdf]

Supporting Information for

In Situ Formation of a Relatively Transparent Ion-Associate Liquid Phase from an Aqueous Phase and Its Application to Microextraction/High-Performance Liquid Chromatography–Fluorescence Detection of Bisphenol A in Water

Noriko Hata *, Seira Takahashi, Sachiko Osada, Sakura Katagiri, Mayumi Naruse, Akane Igarashi, Kazuto Sazawa, Shigeru Taguchi and Hideki Kuramitz

Major of Earth, Life and Environmental Science, Graduate School of Science and Engineering, University of To-yama, 3190 Gofuku, Toyama 930-8555, Japan; sazawa@sci.u-toyama.ac.jp (K.S.); crewoftaguchiut@gmail.com (S.T.); kuramitz@sci.u-toyama.ac.jp (H.K.)

* Correspondence: hata1000ko@gmail.com

The calibration curve for bisphenol A(BPA) from the first and second extractions in IALP (EHOPA+/DS^{-}) were shown in Figure S1.

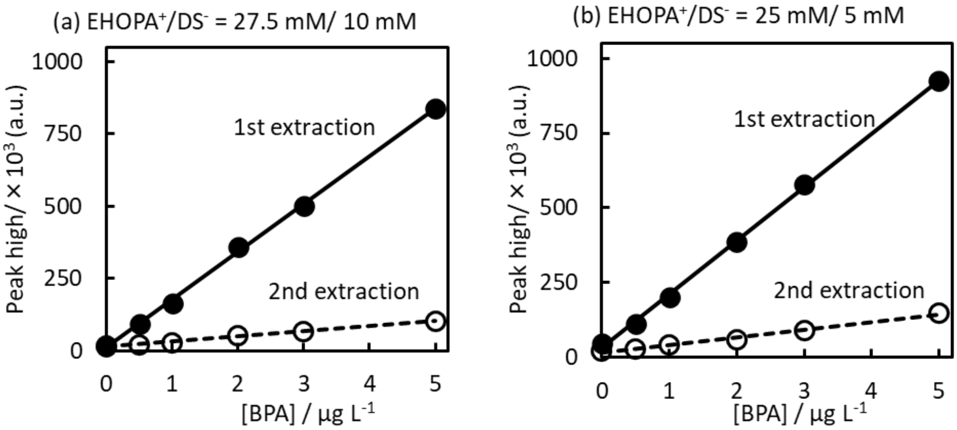

Figure S1 Calibration curve for bisphenol A(BPA) from the first and second extractions in IALP ($EHOPA^{+}$/DS^{-}). Experimental condition: (a) [$EHOPA^{+}$] = 27.5 × 10^{-3} mol L^{-1}, [DS^{-}] = 10 × 10^{-3} mol L^{-1} and (b) [$EHOPA^{+}$] = 25 × 10^{-3} mol L^{-1}, [DS^{-}] = 5 × 10^{-3} mol L^{-1}. Black circle: calibration curve of 1st extraction, white circle: calibration curve of 2nd extraction. BPA concentrations range from 0 to 5 μg L^{-1}, pH = 2, HPLC-FLD.
